# Impaired Acetylcholine-Induced Endothelium-Dependent Aortic Relaxation by Caveolin-1 in Angiotensin II-Infused Apolipoprotein-E (ApoE^−/−^) Knockout Mice

**DOI:** 10.1371/journal.pone.0058481

**Published:** 2013-03-04

**Authors:** Sai Wang Seto, Smriti M. Krishna, Hongyou Yu, David Liu, Surabhi Khosla, Jonathan Golledge

**Affiliations:** The Vascular Biology Unit, Queensland Research Centre for Peripheral Vascular Disease, School of Medicine and Dentistry, James Cook University, Townsville, Queensland, Australia; University of Southampton, United Kingdom

## Abstract

**Objective:**

The angiotensin II (AngII)-infused apolipoprotein E-deficient (ApoE^−/−^) mouse model is widely used to study atherosclerosis and abdominal aortic aneurysm. An increase in blood pressure has been reported in this model however the underlying mechanism has not been fully explored. In this study, we investigated whether vasomotor dysfunction develops in AngII-infused ApoE^−/−^ mice and the underlying mechanism involved.

**Methods:**

ApoE^−/−^ mice were infused with vehicle (distilled water) or AngII subcutaneously for 14 days. Blood pressure and heart rate were measured using the non-invasive tail cuff method. Aortic vascular reactivity and expression of key proteins (endothelial nitric oxide synthase (eNOS), phospho-eNOS and caveolin-1) were assessed using tension myography and Western blotting respectively. Plasma nitric oxide (NO) level was estimated using a colorimetric assay.

**Results:**

AngII infusion caused a time-dependent increase in blood pressure (P<0.001). Aortas from AngII-infused mice were significantly less responsive to acetylcholine-induced endothelium-dependent relaxation when compared to aortas from mice infused with vehicle control (P<0.05). Contractile responses to phenylephrine (P<0.01) and potassium chloride (P<0.001) were significantly enhanced in aortas from AngII-infused mice. eNOS phosphorylation was significantly decreased in the aorta of AngII-infused mice (P<0.05). Aortic caveolin-1 protein expression was significantly increased in AngII-infused mice (P<0.05). Plasma nitrate/nitrite level was significantly reduced in AngII-infused mice (P<0.05). Pharmacological disruption of caveolae using methyl-β-cyclodextrin (MβCD) in isolated aortas from AngII-infused mice caused a significant leftward shift of the acetylcholine-induced relaxation concentration-response curve when compared to vehicle control (P<0.05).

**Conclusion:**

Upregulation of caveolin-1 protein expression and reduced NO bioavailability contributes to aortic endothelial dysfunction in AngII-infused ApoE^−/−^ mice.

## Introduction

Endothelial dysfunction is a common finding in patients with atherosclerosis, abdominal aortic aneurysm (AAA) and hypertension [1], [2]. Nitric oxide (NO) is a key regulator of normal endothelial function [3]. NO is generated by endothelial nitric oxide synthase (eNOS) by catalytic conversion of L-arginine upon receptor activation (e.g. of the muscarinic receptor) or by mechanical forces (e.g. by shear stress) [4], [5]. eNOS is constitutively expressed in endothelial cell and accumulating studies have suggested that various cardiovascular risk factors such as diabetes mellitus, aging and hypertension can impair endothelial function and inhibit the NO signalling pathway [1], [2], [6]. In addition, impaired acetylcholine-induced endothelium-mediated aortic vasodilatation and reduction of NO bioavailability have been demonstrated during hypercholesterolemia in both animal and human studies [7]–[9], suggesting an important role of NO in dyslipidemia-induced vascular dysfunction.

Apolipoprotein E-deficient (ApoE^−/−^) mice are one of the most widely used animal model of atherosclerosis and abdominal aortic aneurysm (AAA) [10]–[12]. These mice develop hypercholesterolemia and aortic plaques when fed normal diet [13] and accelerated atherosclerosis when fed a high fat western-type diet [14]. It is now widely accepted that endothelial dysfunction is one of the early steps in atherosclerosis and AAA [1], [6] and altered NO signalling is a common feature observed in these animal models [7], [14]. Indeed, impairment of endothelium-mediated vasorelaxation in response to acetylcholine has been demonstrated in the aorta of ApoE^−/−^ mice fed a western-type diet [7], [14]. It is interesting to note that when fed a normal diet [15] endothelium-dependent relaxation remains normal up to 6 months of age in ApoE^−/−^ mice. At older ages endothelial dysfunction is correlated with the development and size of aortic plaques [13]. These findings suggest that the endothelial dysfunction is not simply mediated by hypercholesterolemia alone but likely involves additional mechanisms.

Angiotensin II (AngII) infusion is commonly used to promote atherosclerosis and AAA in ApoE^−/−^ mice [12]. We have recently demonstrated that fenofibrate suppressed aortic dilatation and atherosclerosis via increasing eNOS activity in the AngII-infused mouse model [1], suggesting an important role of eNOS activity in this model. Although an increase in blood pressure has been reported in AngII-infused ApoE^−/−^ mice [16], impairment of endothelium-mediated relaxation and the underlying mechanism involved has not been fully explored in this mouse model.

eNOS activity is tightly controlled by various membrane bound receptors and regulatory proteins under physiological conditions [3]. Alternation of these receptors or regulatory proteins can upset the balanced generation of NO. Caveolae are 50–100 nm cell surface plasma membrane invaginations which are abundant in endothelial cells [17]. It has been suggested caveolae play an essential role in regulating NO production by interaction of eNOS and caveolin-1 (Cav-1), a structural protein of caveolae [18]. Pharmacological disruption of caveolae has been shown to impair NO- and endothelium-derived hyperpolarizing factor (EDHF)-mediated acetylcholine-induced vasodilatation in isolated rat blood vessels [18]. Cav-1 has been associated with vascular diseases in both human and animal studies [2], [19]. In Cav-1^−/−^ ApoE^−/−^ mice, atherosclerotic plaque area is markedly reduced despite the presence of hypercholesterolemia [20]. Expression of Cav-1 mRNA and protein were reported to be significantly upregulated in diabetic mice and endothelium-dependent relaxation was markedly suppressed [2]. In addition, it has been suggested recently that amlodipine, an L-type calcium channel blocker, can offer additional cardiovascular protective effects via enhancement of NO production in endothelial cells by antagonising the eNOS/Cav-1 signalling complex [21]. These studies have provided evidence that eNOS/Cav-1 interactions could be a novel target site for therapy of cardiovascular diseases.

We hypothesized that AngII-infusion impaired vascular endothelium function was associated with altered Cav-1 expression in the aortas of ApoE^−/−^ mice. In this study we investigated whether aortic vascular reactivity was altered in AngII-infused ApoE^−/−^ mice by measuring aortic contraction and relaxation in isolated thoracic aortas of AngII-infused ApoE^−/−^ mice. We also assessed eNOS activity and Cav-1 expression in this animal model.

## Materials and Methods

### Animals and Ethics Statement

This investigation conformed to the Guide for the Care and Use of Laboratory Animals published by the US National Institute of Health (NIH Publication No. 85-23, revised 1996). The protocol was approved by the Animal Ethics Committee of James Cook University (Approval Number: A1671). All surgery was performed under intraperitoneal injection of ketamine and xylazine anesthesia, topical application of local anaesthetic (Elmar) was provided as post-operative analgesia and the animals were allowed to recover under supplemental heat using a warming pad. Male ApoE^−/−^ mice (Animal Resources Centre, Western Australia) were housed under a 12∶12-h light-dark cycle (relative humidity: 55–60%; temperature: 22±1°C) and were given standard chow and water ad libitum. At 6 months of age mice were anaesthetised by intraperitoneal injection of ketamine (150 mg/kg) and xylazine (10 mg/kg). Osmotic minipumps (Model 2004, ALZET, USA) were placed into the subcutaneous space along the dorsal midline to deliver 1 µg/kg/min of AngII (Sigma-Aldrich, Castle Hill, Australia) dissolved in distilled water over 14 days. Age-matched ApoE^−/−^ mice in which osmotic pumps delivered distilled water served as vehicle controls. Mice were maintained on a normal laboratory diet throughout the infusion period. After the 14 day infusion, mice were sacrificed by CO_2_ asphyxiation, blood was collected by cardiac puncture and the aortas were isolated for further assessments.

### Non-invasive Tail-cuff Blood Pressure and Heart Rate Measurement

Blood pressure and heart rate were measured at day 0 (baseline), day 7 and day 14 (endpoint) of the experiment using a computerized, non-invasive, tail-cuff system (Kent Scintific, USA). Animals were habituated to the device before measuring the pressures to ensure accurate measurements. Good reproducibility of this technique has been established previously (mean of absolute difference for heart rate: 21.1 beats per minute, 95% confidence interval (CI): 14.8–27.5 and mean of absolute difference for mean blood pressure: 12.3 mmHg, 95% CI: 8.4–16.1).

### Isometric Tension Measurement

Thoracic aortas (length: ∼12 mm; outer diameter ∼0.8 mm) were dissected from the mice immediately after sacrifice. Fat and connective tissues around the vessels were carefully removed under the dissecting stereo-microscope (Leica, Germany). Myograph studies were performed in a 5 mL small vessel wire myograph containing physiological salt solution with the composition (mM) of NaCl 118, CKl 4.7, MgSO_4_ 1.2, KH_2_PO_4_ 1.2, NaHCO_3_ 25, glucose 11 and CaCl_2_ 1.8, as previously described [2]. Two types of experiments were carried out: contraction of vessels and relaxation of precontracted vessels.

In contraction studies, concentration-response curves in response to high [K^+^]_o_ (10–60 mM) and phenylephrine (0.1 nM–3 µM) were constructed. Increasing concentrations of individual contractile agents were administrated at half-log increments, and the response at each concentration of drug added (expressed as the final bath concentration) was measured using the MacLab Chart v 7.2.1 programme (AD Instruments, Australia).

In relaxation studies, to exclude the involvement of the cyclo-oxygenase cascade and acetylcholinesterase, indomethacin (1 µM, a selective cyclo-oxygenase inhibitor) and neostigmine (10 µM, an acetylcholinesterase inhibitor) were included in the Krebs’ solution in all experiments. In some preparations, the role of NOS and caveolae were evaluated by using *N* ^ω^-nitro-l-arginine methyl ester (l-NAME) (20 µM, a NOS-inhibitor) and methyl-β-cyclodextrin (1 mM, a cholesterol-extracting agent), respectively. All blockers used in this study were allowed to incubate with the preparation for 30 min before the construction of a concentration–response curve. Increasing concentrations of individual relaxants were administered at half-log increments at the plateau (i.e. steady-state) of the previous response. The response at each concentration of drug added (expressed as final bath concentration) was measured using the MacLab Chart v 7.2.1 programme (AD Instruments, Australia). Relaxation in response to individual agents was expressed as % of the phenylephrine (1 µM) contraction and 100% relaxation was considered when the active tone returned to the baseline level.

### Plasma Nitrite/nitrate and Cholesterol Measurement

Measurements of total plasma cholesterol, high density lipoprotein (HDL) and low density/very low density lipoprotein (LDL/VLDL) were performed in plasma in duplicate using a commercial kit in accordance with the manufacturers’ instructions (inter-assay coefficient of variation 3.4%) (Abcam). Cholesterol oxidase specifically recognizes free cholesterol and produces products which react with a probe to generate fluorescence (Ex/Em = 538/587 nm) and were measured using an Omega plate reader.

Total nitrate was measured in plasma samples by a nitrate/nitrite colorimetric assay kit following manufacturer’s protocol (inter-assay coefficient of variation 2.7%) (Cayman). Briefly, nitrate was converted to nitrite using nitrate reductase. Subsequently addition of the Griess reagents converted nitrite into a deep purple azo compound and the absorbance was measured at 540 nm using an Omega plate reader.

### Western Blotting

Thoracic aortas were homogenized in RIPA buffer (Thermo Scientific, USA) in the presence of protease inhibitors (Roche Applied Science, USA) to obtain extracts of proteins. Protein concentrations were determined using the Bradford protein assay kit (BioRad, USA). Samples (25 µg of protein per lane) were loaded onto a 10% SDS-polyacrylamide gel electrophoresis gel. After electrophoresis (110 V, 90 min), the separated proteins were transferred (15 mA, 60 min) to polyvinylidene difluoride membranes (BioRad, USA). Non-specific sites were blocked with 5% non-fat dry milk in TBSt (BioRad, USA) for 60 min, and the blots were then incubated with anti-phospho-eNOS (Ser 1177) antibody, 1∶1000 (Cell Signalling, USA); anti-eNOS, 1∶1000(Cell Signalling, USA); or anti-Cav-1, 1∶1000 (Santa Cruz, USA) in 5% non-fat milk (BioRad, USA) overnight at 4°C. Anti-rabbit HRP conjugated IgG, 1∶1000 (DakoCytomation, Denmark) in 5% non-fat milk (60 min, room temperature) was used to detect the binding of its correspondent antibody. Membranes were stripped with stripping solution (Thermo Scientific, USA) for 20 mins at room temperature and re-blotted with anti-β actin antibody, 1∶6000 (Sigma-Aldrich, USA) in 5% non-fat milk (60 min, room temperature) to verify equal loading of protein in each lane. The protein expression was detected with Western Lightning Chemiluminescence Reagent Plus (PerkinElmer Life Sciences, USA) and quantified by Quantity One (version 4.6.7) software (BioRad, USA).

### Statistical Analysis

Data were presented as mean ± SEM of *n* experiments. Statistical comparisons were performed using t-test or two-way analysis of variance (ANOVA), where appropriate. Differences were considered to be statistically significant at *P*<0.05. All statistical analysis was performed using GraphPad Prism 5 software (GraphPad Software, Inc., USA).

## Results

### Effect of AngII Infusion on Blood Pressure and Heart Rate in ApoE^−/−^ Mice

**Figure 1:**
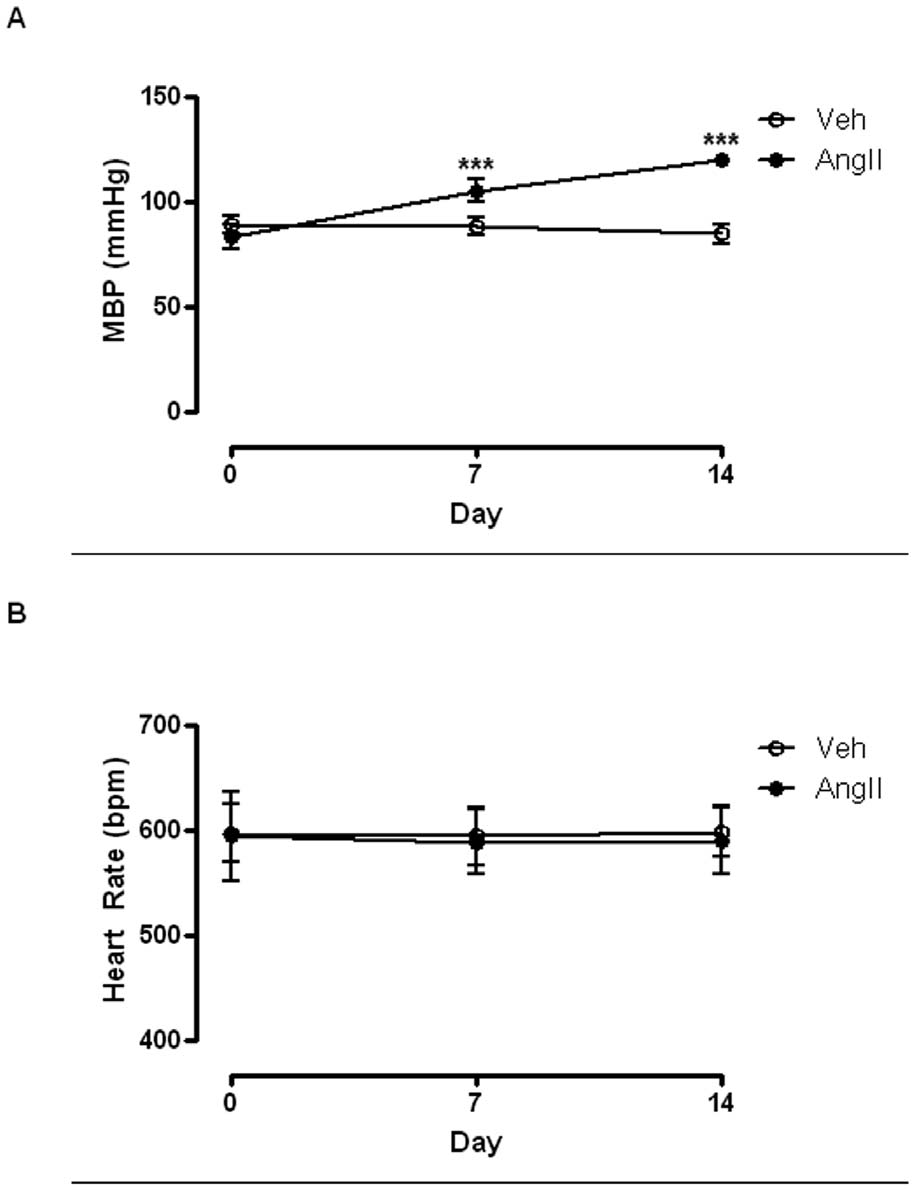
AngII infusion increases mean blood pressure in ApoE^−/−^ mice. Effects of AngII infusion on (A) heart rate and (B) mean blood pressure (MBP) in ApoE^−/−^ mice. Results are mean±SEM (n = 6); Statistical significance shown as ***P<0.0001.

AngII infusion resulted in an increase in mean blood pressure (MBP) in ApoE^−/−^ mice in a time-dependent manner. MBP was significantly higher in the AngII-infused group when compared with the vehicle-infused group as measured at day 7 (Control vs AngII: 88.13±1.81 mmHg vs 104.90±2.20 mmHg) (n = 6; *P*<0.001) and day 14 (Control vs AngII: 84.33±1.86 mmHg vs 119.40±1.29 mmHg) (n = 6; *P*<0.001) (Figure 1A). Heart rate was not altered by either AngII or vehicle infusion in ApoE^−/−^ mice throughout the 14-day period (Control vs AngII at day 14: 597.10±9.74 bpm vs 590.10±13.39 bpm) (n = 6; *P*>0.05) (Figure 1B).

### Effect of AngII Infusion on Vascular Relaxation of Isolated Thoracic Aortas from ApoE^−/−^ Mice

**Figure 2:**
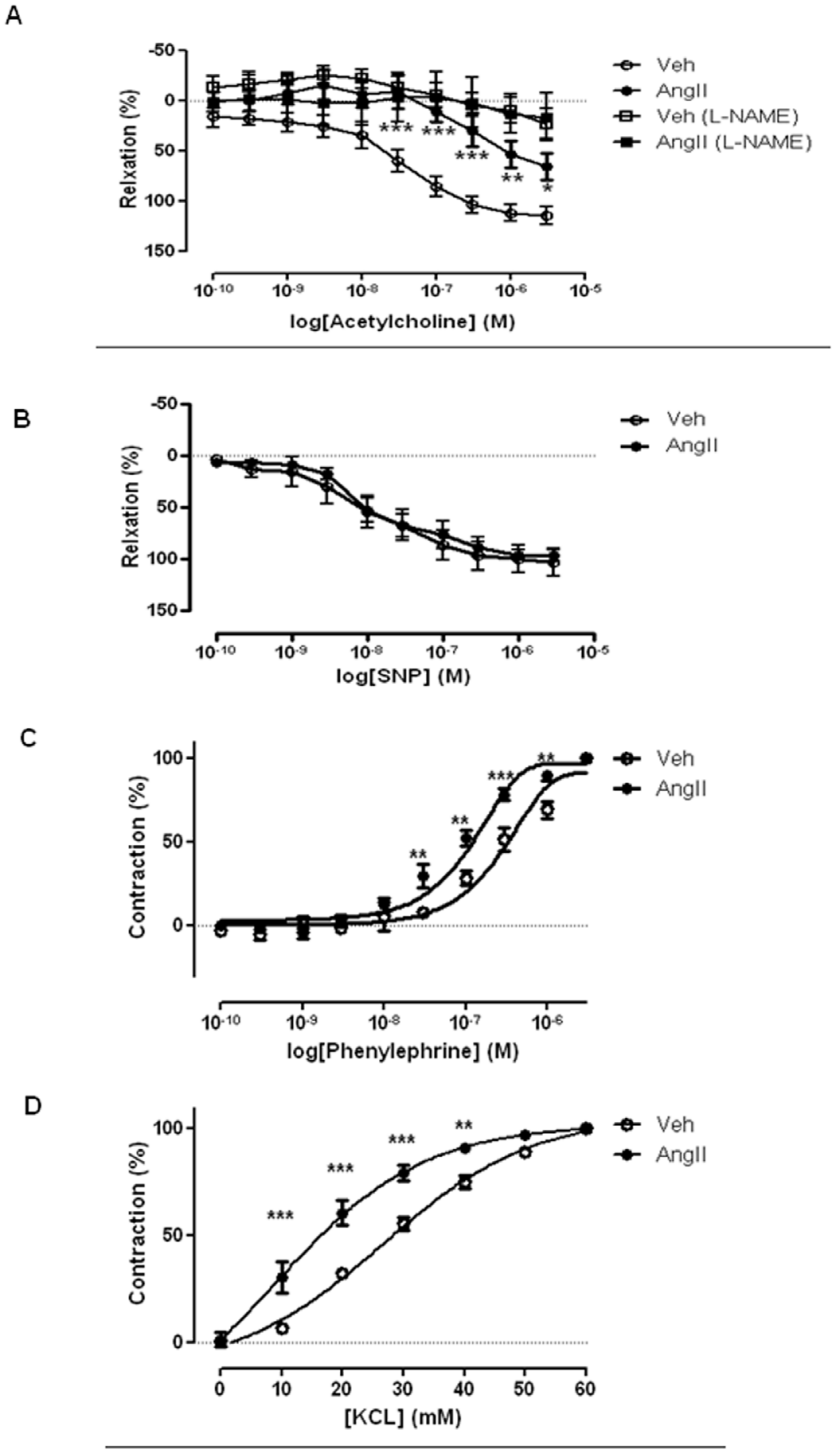
AngII infusion alters aortic relaxations and contractions in ApoE^−/−^ mice. (A) Concentration-response curve of endothelium-dependent relaxation to acetylcholine, with or without the present of L-NAME (20 µM) and (B) endothelium-independent relaxation to SNP in aortas of saline-infused (Veh) and AngII-infused (AngII) ApoE^−/−^ mice. (C) Concentration-response curve to phenylephrine- and (D) KCl-induced contraction in aortas of saline-infused (Veh) and AngII -infused (AngII) ApoE^−/−^ mice. Results are mean±SEM; (n = 5–6); Statistical significance shown as *P<0.05; **P<0.01 and ***P<0.0001.

Basal tone of the rings were similar in aortas from the vehicle- and AngII-infused mice (Control: 8.83±0.84 mN; AngII: 8.52±0.34 mN) (P>0.05; n = 6). In aortas from vehicle-infused ApoE^−/−^ mice acetylcholine (0.1 nM –3 µM) caused a concentration-dependent relaxation of the isolated thoracic aorta with 100% relaxation at ∼1 µM (Figure 2A). The acetylcholine-induced relaxation of aortas from AngII -infused mice was significantly smaller than that observed in aortas from control mice. There was a marked rightward shift of the concentration-response curve to acetylcholine (EC_50_: Control: 26.3±2.09 nM; AngII: 798.7±1.40 nM) and a reduced maximum relaxation response at 3 µM (Control: 114.6±8.93; AngII: 65.98±13.33) (n = 5–6; *P*<0.05) (Figure 2A). In addition, the acetylcholine-induced relaxation was greatly suppressed by L-NAME (20 µM) in aortas from both vehicle- and AngII -infused mice (Figure 2A). Cumulative administration of sodium nitroprusside (SNP) (0.1 nM–3 µM) caused a concentration-dependent relaxation of isolated thoracic aortas from both vehicle- and AngII -infused mice. No significant difference was observed in the SNP-induced relaxation of aortas from the two groups (Figure 2B).

### Effect of AngII Infusion on Vascular Contraction of Isolated Thoracic Aortas from ApoE^−/−^ Mice

Cumulative administration of phenylephrine (0.1 nM- 3 µM) caused a concentration-dependent contraction of isolated thoracic aortas from vehicle-infused ApoE^−/−^ mice. A significant leftward shift of the concentration-response curve to phenylephrine was observed in the isolated aortas from the AngII-infused mice (EC_50_: Control: 294.8±5.18 nM; AngII: 83.9±4.15 nM) (*P*<0.01; n = 5–6) (Figure 2C). Similarly, potassium chloride (10–60 mM), caused a concentration dependent contraction of the isolated aortas of ApoE^−/−^ mice with a significant leftward shift of the concentration-response curve observed in AngII-infused mice when compare with the vehicle-infused animals (EC_50_: Control: 26.67±2.90 mM; AngII: 15.27±4.89 mM) (*P*<0.0001; n = 5–6) (Figure 2D).

### Effect of AngII Infusion on Cav-1, p-eNOS(Ser1177) and Total eNOS Protein Expression in the Thoracic Aortas of ApoE^−/−^ Mice

**Figure 3:**
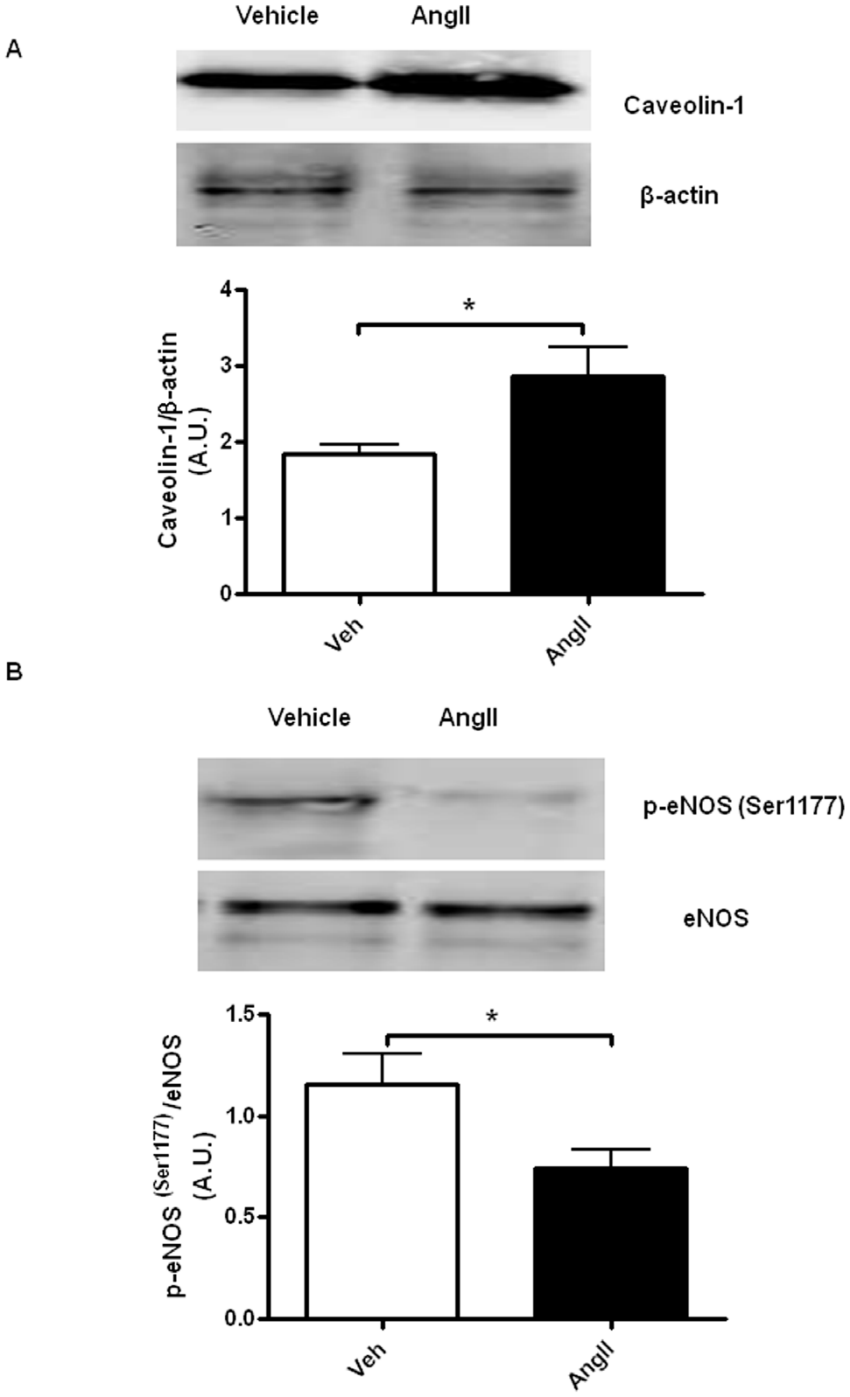
AngII infusion alters aortic Cav-1 and phospho-eNOS expressions. (A) Representative Western blot analysis of caveolin-1 protein expression in aortas of saline-infused (Veh) and AngII-infused (AngII) ApoE^−/−^ mice. Bar chart indicates results of relative densitometry analysis of caveolin-1 protein relative to β-actin. (B) Representative Western blot analysis of phospho-eNOS (Ser1177) and total eNOS protein expression in aortas of saline-infused (Veh) and AngII -infused (AngII) ApoE^−/−^ mice. Bar chart indicates results of relative densitometry analysis of p-eNOS protein relative to total eNOS. Results are mean±SEM; n = 6; Statistical significance shown as *P<0.05.

Protein expression of Cav-1, p-eNOS and total eNOS in the isolated thoracic aortas were evaluated and compared between the vehicle-infused and AngII-infused ApoE^−/−^ mice. Our results revealed that there was significant upregulation of Cav-1 protein expression in aortic sample from AngII-infused mice (n = 6; *P*<0.05) (Figure 3A). Expression of p-eNOS (Ser1177) and total eNOS protein in the isolated aortas from the two groups of mice were compared. Expression of total eNOS was similar in the aortas of saline- and AngII mice. A marked reduction of p-eNOS(Ser1177) expression was observed in aortas from the AngII-infused mice when compared to the vehicle-infused mice (n = 6; *P*<0.05) (Figure 3B).

### Effect of AngII Infusion on Plasma Nitrite/nitrate, Total Cholesterol, High Density Lipoprotein (HDL) and Very Low Density Lipoprotein (VLDL) in ApoE^−/−^ Mice

**Figure 4:**
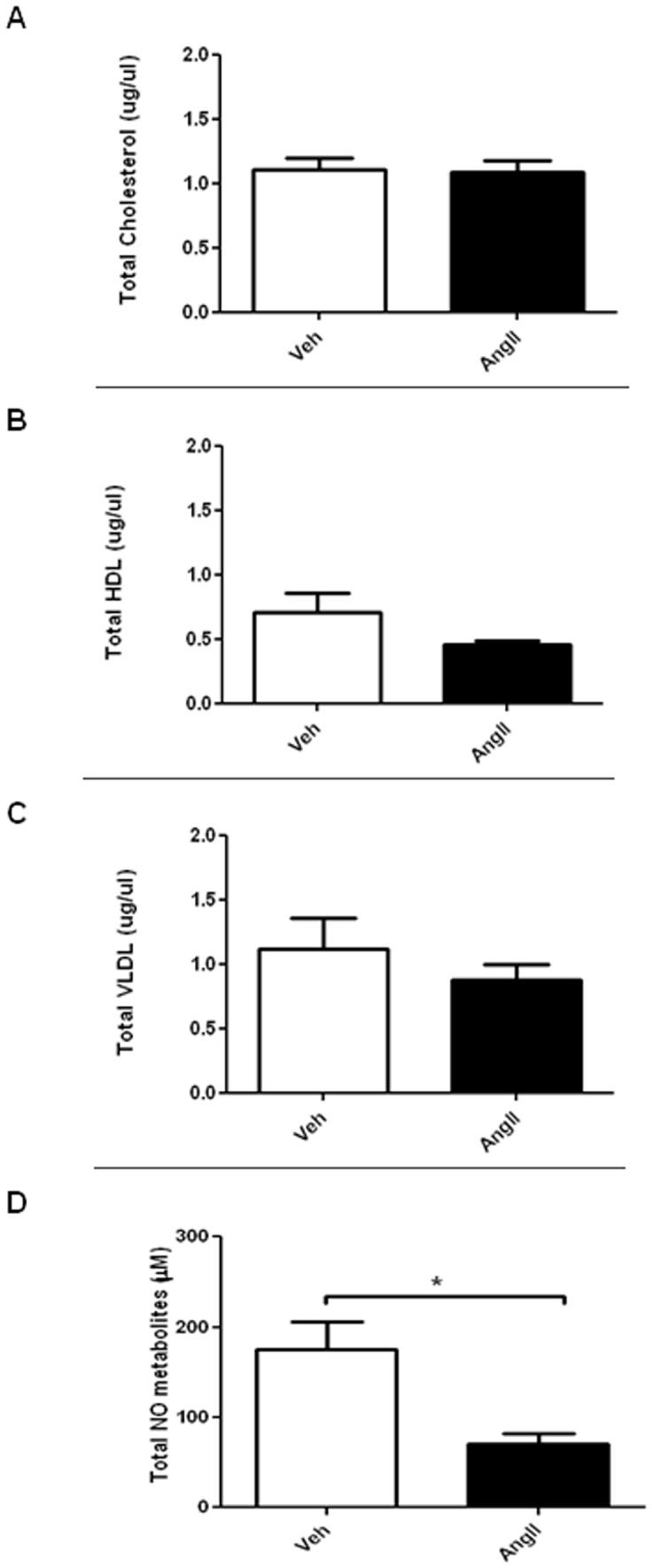
Effects of AngII infusion on plasma nitrite/nitrate, total cholesterol, high density lipoprotein (HDL) and very low density lipoprotein (VLDL). Box plots showing plasma (A) total cholesterol; (B) HDL, (C) VLDL and (D) total NO metabolites in saline-infused (Veh) and AngII -infused (AngII) ApoE^−/−^ mice. Results are mean±SEM; n = 5; Statistical significance shown as *P<0.05.

Plasma total cholesterol, HDL and VLDL were not significantly different between vehicle- and AngII-infused mice (Figure 4A, B and C). Total plasma nitric oxide metabolite (nitrite/nitrate) level was significantly lower (∼2 fold) in AngII-infused mice (n = 5; *P*<0.05) (Figure 4D).

### Effect of Methyl-β-cyclodextrin on Acetylcholine-induced Relaxation in Isolated Aortas from Vehicle- and AngII-infused ApoE^−/−^ Mice

**Figure 5:**
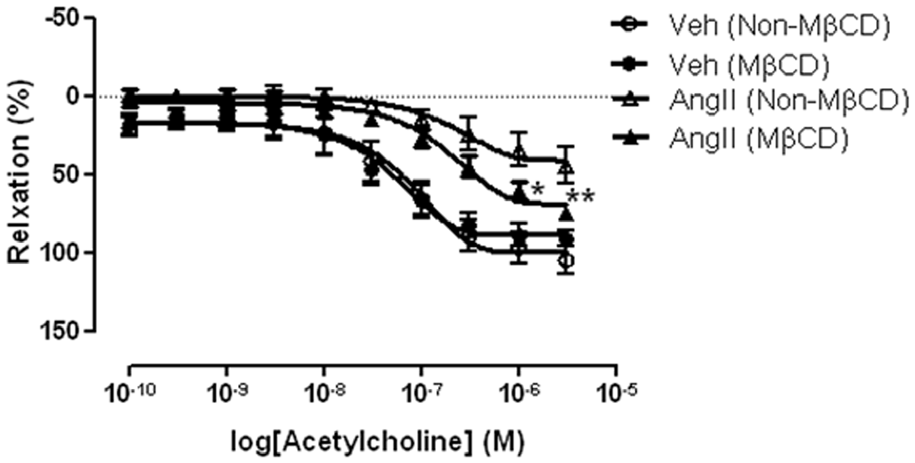
Effects of chemical disruption of caveolae on acetylcholine-induced aortic relaxation. Concentration-response curve of endothelium-dependent relaxation to acetylcholine in aortas of vehicle- and AngII -infused ApoE^−/−^ mice with or without the presence of methyl-β-cyclodextrin (MβCD) (1 mM). Results are mean±SEM and expressed as percentage of relaxation of the submaximal contraction of phenylephrine (1 µM) (n = 5); Statistical significance shown as *P<0.05 and **P<0.01.

The effect of chemical disruption of caveolae on acetylcholine-induced relaxation was studied in isolated aortas of vehicle- and AngII-infused mice. The isolated aortas were preincubated either with methyl-β-cyclodextrin (MβCD) or without (non-MβCD) for 60 minutes prior to construction of concentration-response curves. Neither MβCD or non- MβCD pre-incubation influenced baseline tone at any time. In the presence of MβCD, the acetylcholine-induced relaxation in isolated aortas from AngII-infused mice was significantly greater at 3 µM (Control: 44.72±11.62; MβCD: 75.07±4.07) (P<0.05; n = 5) with a leftward shift of the concentration-response curve when compare with the non-MβCD treated aortas. However, MβCD did not alter acetylcholine-induced relaxation in isolated aortas from the vehicle-infused mice (P>0.05; n = 5) (Figure 5).

## Discussion

This study demonstrates for the first time that an increase in caveolin-1 protein expression is associated with the impairment of acetylcholine-induced endothelium-dependent aortic relaxation found in AngII-infused ApoE^−/−^ mice. It is well known that malfunction of the endothelium is a hallmark of vascular diseases seen in patients with hypertension and atherosclerosis. Our results suggest that upregulation of caveolin-1 protein expression and reduced NO bioavailability contributes to aortic endothelial dysfunction in AngII-infused ApoE^−/−^ mice.

Classically, relaxation of blood vessels by acetylcholine is mediated by NO generated from eNOS and activation of soluble guanylyl cyclase [2]. Previous studies have demonstrated that acetylcholine, but not DEA-NONOate (a NO-donor), induced vascular relaxation was reduced in hypercholesterolemic ApoE^−/−^ mice [22], [23]. Similarly, in this study, we observed a marked decrease in acetylcholine-induced aortic relaxation in AngII-infused ApoE^−/−^ mice suggesting reduction of eNOS activity. It is important to note that the acetylcholine-induced relaxation was substantially inhibited by L-NAME, an eNOS inhibitor, confirming that the relaxation was mediated by eNOS-derived NO. Although altered responsiveness to NO in vascular smooth muscle cells (VSMCs) has been observed in ApoE^−/−^ mice [7], the preserved SNP-induced endothelium-independent relaxation observed in our current study suggests the impaired relaxation was not a consequence of changes in soluble guanylyl cyclase activity and/or subsequent cGMP formation in VSMCs. An enhanced vasoconstriction response to α-adrenoceptor agonist without alteration in endothelium function was previously reported in ApoE^−/−^ mice [24]. We observed that the increased vasoconstriction response was not limited to an α-adrenoceptor agonist (ie. receptor-mediated response), but was also demonstrated in response to KCl (i.e. non-receptor-mediated response) in AngII-infused mice. The possible explanation for this alteration in both receptor-mediated and non-receptor-mediated vasoconstriction is that endothelial NO is basally released as a buffering mechanism limiting the magnitude of vascular contraction response under normal physiological conditions [23], [25]. In the case of endothelial dysfunction, this balance could be upset resulting in an enhanced contraction response. In agreement with this hypothesis, we noticed a significant reduction in plasma nitrite and nitrate levels in AngII-infused mice. A reduced phenylephrine-induced aortic contraction was reported in hypercholesteremic rabbits in association with an alteration of the cyclooxygenase (COX) signalling pathway [26]. In this study, plasma cholesterol level was not different between the AngII- and vehicle- infused mice, minimizing the possibility that the observed responses were mediated by hypercholesteremia-altered COX signalling. Collectively, these data support the idea that altered vascular reactivity in AngII infused ApoE^−/−^ mice is mediated by impaired endothelial function.

Phosphorylayion of eNOS at the Ser1177 residue is one of the major steps in eNOS activation [3]. The decreased ratio of p-eNOS to eNOS in the thoracic aortas of AngII-infused mice observed in our study suggests a decreased efficiency of eNOS activation by a mechanism which limits the phosphorylation of this enzyme at the Ser1177 site. Numerous studies have suggested that eNOS can directly interact with the scaffolding domain of Cav-1, a major structural protein of caveolae [17], [27]. Increased NO production and reduced arterial myogenic tone have been observed in Cav-1 knockout mice. These studies clearly indicate an essential role of Cav-1 in the negative regulation of eNOS activity [17], [28]. Furthermore, increased Cav-1 mRNA and protein expression have been observed in the cardiovascular preparations of diabetic obese mice [2] and in rats received high salt intake [29], in association with impaired eNOS activity. These reports further support the idea that altered expression of Cav-1 could be associated with endothelial dysfunction in vascular diseases. Our results demonstrate for the first time that aortic Cav-1 protein expression was markedly upregulated in ApoE^−/−^ mice receiving AngII infusion. Bodor et al have recently shown that AngII infusion increases permeability of human cultured umbilical vein endothelial cells (HUVECs) by upregulation of PV-1 protein expression and increased caveolae accumulation at cell membranes and within the cytoplasm [30]. These results further strengthen the idea that AngII could contribute to the regulation of Cav-1 expression and/or caveolae formation. Finally, we provided additional evidence for the role of Cav-1/caveloae in the reduced endothelium-dependent acetylcholine-induce aortic relaxation in AngII-infused ApoE^−/−^ mice by disrupting caveolae and membrane fluidity pharmacologically [31]. Methyl-β-cyclodextrin (MβCD), a cholesterol-binding agent, partially restored the acetylcholine-induced aortic relaxation in the AngII-infused mice, indicating the importance of caveolae and functional properties of membranes in acetylcholine-mediated eNOS signalling transduction. Although, most previous reports demonstrated a reduced relaxant response to acetylcholine after MβCD treatment, it is important to point out that, responses observed from those studies were conducted on vascular preparations from animals which were free from AngII treatment and/or diseases [18], [31]. Interestingly, a recent study by Burger et al demonstrated that AngII induced hypertension in ApoE^−/−^ mice involves microparticle formation and increased endothelial superoxide generation where release of the microparticles were blocked by membrane/caveloae disruption using MβCD [32]. Hence, it is possibly that the enhanced endothelium-dependent response in our study was mediated by both Cav-1/eNOS interaction and inhibition of endothelial-derived microparticle generation.

The AngII-infused ApoE^−/−^ mouse model used in this study is a widely used animal model in atherosclerosis and aneurysm research [12]. Consistent with previous reports [16], the present study demonstrated that blood pressure was elevated in a time-dependent manner in ApoE^−/−^ mice infused with AngII. The magnitude of the blood pressure elevation (∼35 mmHg) in our study was ∼10 mmHg higher when compared to that previously shown [16]. The use of ApoE^−/−^ mouse of older age (6 months vs 2 months) in this study could have contributed to this discrepancy, since enhanced blood pressure and sensitivity to AngII in aged ApoE^−/−^ mice have been previously reported [33]. Previous studies have suggested that the elevation of blood pressure by AngII infusion is simply due to the vasoconstriction property of AngII and independent from AAA and atherosclerosis [16]. It is interesting to point out that, our current results clearly demonstrated a reduced endothelium-dependent aortic relaxation in the AngII-infused mice indicating AngII infusion could alter vascular reactivity beyond its vasoconstriction property. Moreover, recent studies from our group and others have demonstrated the important role of eNOS activity in AAA and atherosclerosis in the AngII-infused mouse model [1], [6], suggesting a possible link between AngII and the pathogenesis of diseases involving endothelium dysfunction. A recent clinical report has suggested that arterial diseases are associated with disturbed interaction between Cav-1 and eNOS [19]. Finally, previous studies have suggested that upregulation of cell adhesion molecules expression, such as ICAM -1 and VCAM-1, play an important role in atherosclerosis development [34], [35]. Endothelium dysfunction, including reduced eNOS activity, is closely associated with the enhanced endothelial ICAM-1 expression in ApoE^−/−^ mice [36]. Although this is not explored in the current study, it is logical to hypothesize that AngII-induced atherosclerosis in ApoE^−/−^ mouse is, at least, partly mediated by the increased Cav-1-mediated suppression of eNOS activity. Putting all these together, our results highlight the possible important role of Cav-1 in endothelium function in AngII-infused ApoE^−/−^ mice. This finding may allow for the development and investigation of new drugs for cardiovascular conditions by targeting Cav-1.
